# Selection of patient reported outcomes questions reflecting symptoms for patients with metastatic melanoma receiving immunotherapy

**DOI:** 10.1186/s41687-019-0111-8

**Published:** 2019-03-21

**Authors:** Lærke K. Tolstrup, Lars Bastholt, Ann-Dorthe Zwisler, Karin B. Dieperink, Helle Pappot

**Affiliations:** 1 Department of Oncology, Odense University Hospital, Odense, Denmark; 2 REHPA –The Danish Knowledge Center for Rehabilitation and Palliative Care, University of Southern Denmark, Odense, Denmark; 3 Institute of Clinical Research, University of Southern Denmark, Odense, Denmark; 4 Department of Oncology, Copenhagen University Hospital, Rigshospitalet, Copenhagen, Denmark

**Keywords:** Patient-reported outcomes, PRO-CTCAE, Item-selection, Symptomatic toxicity, Adverse events, Melanoma, Immunotherapy

## Abstract

**Context:**

Toxicity-monitoring plays an important role in all cancer treatment, however, early recognition is vital for detecting and treating immune-related symptoms. Preparing a Patient Reported Outcomes tool and including melanoma patients receiving immunotherapy in the reporting of symptoms, may optimize toxicity-monitoring.

**Objectives:**

The objective of this study was to identify the symptoms and their equivalent questions to include from the Patient-Reported Outcomes Common Terminology Criteria for Adverse Events (PRO-CTCAE) library for melanoma patients, receiving immunotherapy and, further, to evaluate if all relevant symptoms are covered by this tool.

**Methods:**

To establish the relevant symptoms, three measures were taken. First, a literature search was carried out in three databases. Second, a chart audit was performed including medical records from melanoma patients receiving immunotherapy. Finally, the product information for the relevant immunotherapies was studied.

**Results:**

Ten articles were included as a result of the literature search. As for the chart audit, a total of 37 patients (48 treatments with immunotherapy) were included. Overall, the reported symptoms from the literature review aligned with those identified in the chart audit. The examination of the product information supported the findings from review and chart audit, revealing only one additional symptom. In total, 28 PRO-CTCAE symptoms were selected comprising of 56 PRO-questions plus an additional question on blood in stool.

**Conclusion:**

When preparing a Patient Reported Outcomes tool it is important that the preparatory work of selecting questions is done properly. By going through the literature, performing a chart audit, and examining the product information, the most important and relevant symptoms have been uncovered, facilitating the design of a PROquestionnaire, based on PRO-CTCAE, that fits the patient population under investigation.

## Introduction

The number of Danes who are diagnosed with malignant melanoma have increased significantly during the last 50 years. Approximately 2200 new cases are reported every year. Malignant melanoma is the most common cancer form in the 15–34 year old and more than 400 persons are diagnosed with metastatic disease each year [1]. This development aligns with the development worldwide [2]. When metastatic, the majority of patients are treated with immunotherapy, using checkpoint inhibitors either as monotherapy or in combination [3]. Survival has improved significantly with these new treatment strategies. However, the adverse events (AEs) that patients may experience can be severe and potentially life-threatening [4–7]. Studies report that 16% of patients treated with immunotherapy targeting PD-1 experience CTC > = grade 3 AEs measured by the Common Criteria for Adverse Events (CTCAE). With immunotherapy targeting CTLA-4, the number is 27%, and when the drugs are combined, the frequency is 55% [6]. With all cancer drugs, toxicity-monitoring plays an important role, however, early recognition is vital for detecting and treating immune-related AEs. If symptoms are discovered early, relevant treatment can be initiated in time, and major complications avoided [8].

CTCAE is widely used when it comes to toxicity-monitoring in oncological, clinical trials and in routine cancer care. The CTCAE consists of 790 AEs and is divided into three categories: laboratory-based events, physical examination findings and symptomatic adverse events [9]. Physicians perform a systematic evaluation using the CTCAE to describe the severity of organ toxicity for patients receiving cancer therapy. In Denmark, a melanoma patient who receives immunotherapy will be clinically evaluated every third week prior to treatment. Consequently, there is a risk that a symptom may go from mild to severe in the time span. Moreover, evidence suggests that clinicians may underestimate symptom onset and severity compared to patient report [10]. It may be hypothesized that including patients in the reporting of symptoms - and more frequently [11] - can optimize symptom monitoring.

One way to increase patient involvement is to use patient reported outcomes (PROs). Applying a PRO-tool which resembles the well-known CTCAE grading scale seems advantageous. For this purpose, the National Cancer Institute has developed a tool appropriate for patient self-reporting. A total of 78 symptoms, approximately 10%, in the CTCAE guidelines have been found appropriate for self-monitoring and now constitute what has been labeled as the PRO-CTCAE [10, 12]. As each adverse event is elicited using between one to three questions on frequency, severity and interference with daily activities, there are 124 individual questions representing the 78 symptoms. From this question library and its attached form builder, it is possible for researchers and oncologists to choose relevant symptoms and create a questionnaire [13]. The PRO-CTCAE is translated and validated in a Danish version [14], and a Danish feasibility study has recently been carried out [15], demonstrating that the tool is feasible in a prostate cancer population receiving chemotherapy. However, no guidelines exist on how to select the relevant PRO-items representing expected symptoms in different disease and treatment situations.

The advantages of using PROs in cancer treatment and care are debated [16, 17]. Current data suggests that physical symptoms are more likely to improve after PRO interventions compared to quality of life (QoL), supportive care needs or psychological symptoms [16]. More evidence is needed, however, to determine if the implementation of PROs in relation to, for example, symptom reporting is worthwhile. So far, research involving PRO-CTCAE has focused on toxicity monitoring associated with other cancer therapies [10]. The tool has not been reported as used by patients receiving immunotherapy. The pattern of symptoms with this treatment modality differs considerably from the one patients experience when they receive chemotherapy [8]. Thus, it is highly relevant to select the symptoms that fit the toxicity-profile and provide and an unbiased presentation when designing a PRO-tool for melanoma patients receiving immunotherapy [18].

The objective of this study was to identify the symptoms appropriate for patient self-reporting and their equivalent PRO-questions to include from the PRO-CTCAE library for melanoma patients receiving immunotherapy and, further, to evaluate if all relevant symptoms can be covered by the tool.

## Material and methods

To establish the relevant symptoms to include in a subsequent randomized trial, a project group was formed. Besides the project manager, the group consisted of two physicians who were experts in handling immune-related symptoms. Moreover, one had experience with selecting relevant PRO-CTCAE-items. It was decided in advance that due to the purpose of the study i.e. to identify symptoms appropriate for self-reporting, labatory based events and physical examination findings would be excluded during the selection process. Only symptomatic AEs that the patients would meaningfully be able to report were to be included.

### Literature search

A literature search was performed in the three literature databases Pubmed, Embase, and Cinahl in June 2016 using the Boolean logical operators AND/OR to combine the search terms. A combination of keywords for *cancer*, *immunotherapy, adverse events* and *melanoma* was combined. Before doing the final search, the search terms were monitored by an expert on literature searches which resulted in minor changes. Articles were included if the studies described were randomized clinical trials that ^i)^ compared immunotherapy with chemotherapy or placebo, ^ii)^ compared immunotherapy in different doses, ^iii)^ compared different immunotherapies, or ^iv)^ compared immunotherapy with other cancer therapies. Articles not written in English were excluded. Articles were eligible if indexed between January 1, 1996 and June 22, 2016.

First, one reviewer screened the titles and abstracts to eliminate all irrelevant references. Second, another reviewer took part in determining the references relevant for full text review. Hereafter, both reviewers jointly decided which articles should be included.

### Chart audit

In addition to the literature review, a chart audit was performed to examine if the AEs found in the international literature were consistent with symptoms melanoma patients treated with immunotherapy experienced in daily practice. The chart audit was performed at the Department of Oncology at Odense University Hospital in the Region of Southern Denmark. Permission was granted from the head of department. Thirty-seven medical records were examined with oral and written informed consent from patients between June and August 2016. No selection criteria was applied and all melanoma patients treated with immunotherapy were asked to participate. No patients refused, however, due to administrative errors, a few patients were not recruited. The patients included in the chart audit had received either anti-PD-1 or anti-CTLA-4. The AEs identified in the medical records were primarily found in a prespecified toxicity-monitoring form build upon the CTCAE grading scale v4 for physicians to register the severity of AEs. In addition, the free text notes in the medical records were included. If a health professional had noted, for example, that a patient suffered from taste changes, the term was translated into the CTCAE term dysgeusia, making it possible to align all the patients´ AEs.

### Product information

Finally, the product information from the European Medicines Agency (EMA) for Yervoy (Ipilimumab), Keytruda (Pembrolizumab) and Opdivo (Nivolumab) [19] were studied to ensure that no adverse events had been overlooked. If an AE was reported in one of the EMA sources - and not already identified by the two other data souces - it was included if it affected more than 10% of the patients.

After the construction of the PRO-CTCAE questionnaire, the instrument was pilot-tested by four patients and five healthcare professionals to ensure face validity.

## Results

### Literature search

**Fig. 1:**
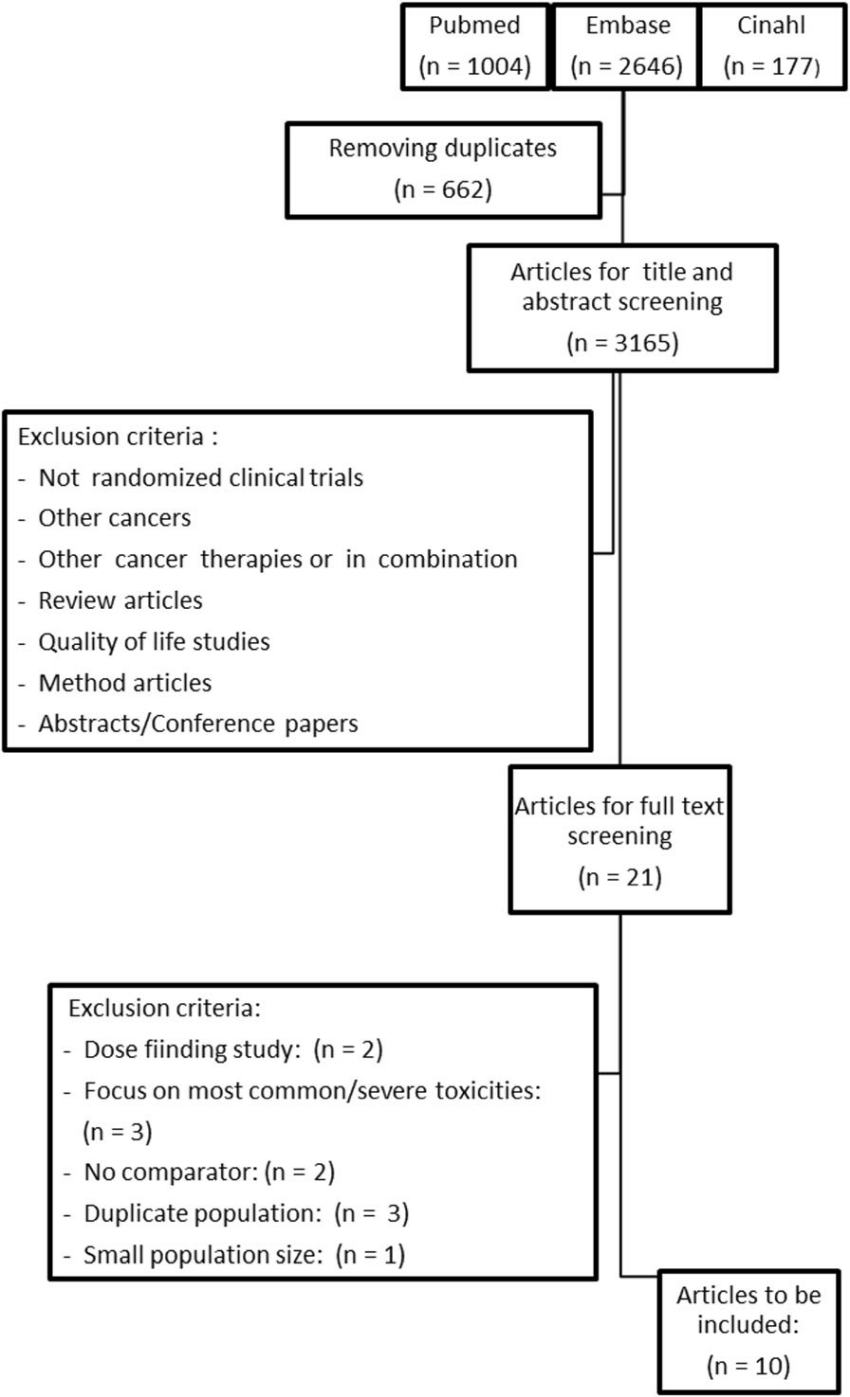
Article selection flowchart. Articles on immune-related adverse events identified in melanoma patients treated with immunotherapy

Initial literature searches retrieved 3.165 titles from the databases. After title and abstract screening and full text screening had been performed, ten articles fulfilled the inclusion criteria and were extracted (Fig. 1). The articles were randomized, clinical trials including a total of 5.706 patients (Table 1). Thus, the number of trial participants were judged sufficient to satisfy the trial objective of identifying relevant AEs. Only two trials had a sample size of less than 400 patients and were not multi-center trials. The immunotherapy in the trials was either compared to placebo, other immunotherapy, or other anti-cancer drugs. The studies tested Ipilimumab [4–6, 20, 21], Pembrolizumab [7, 21, 22] or Nivolumab [6, 23, 24] as monotherapy or a combination [6, 20] of two of the drugs. One study [25] evaluated sequential single-drug therapy with Ipilimumab followed by Nivolumab (or the reverse sequence). One article evaluated Ipilimumab given as adjuvant therapy, whereas the remaining nine were all concerned with treatment of metastatic disease. Some of the articles, including supplementary material, decribed the most common AEs, occurring in at least 5–10% of patients, while others also reported AEs that occurred in as few as 1–2% of the patients.

**Table 1:** Characteristics of studies included to determine which AEs to include in the PRO-CTCAE for melanoma patients

Trial	Design	Enrollment size *N*=	Study drug	Dose (mg/kg)
Eggermont et al. [3]	Adjuvant, Randomized phase 3	951	Ipilimumab	10
Hodi et al. [2]	Phase 3 Randomized	676	Ipilimumab	3
Larkin et al. [4]	Phase 3 randomized	945	Nivolumab Nivolumab/Ipilimumab Ipilimumab	3; 1 + 3; 3
Postow et al. [17]	Double-blind Phase 2 – dose ranging	142	Ipilimumab or Ipilimumab/Nivolumab	3; 3 + 1
Ribas et al. [19]	Phase 1b	655	Pembrolizumab Pembrolizumab	10; 2
Ribas et al. [5]	Phase 2	540	Pembrolizumab Pembrolizumab	2; 10
Robert et al. [20]	Randomized, phase 2	418	Nivolumab	3
Robert et al. [18]	Phase 3	834	Pembrolizumab ipilimunab	10; 3
Weber et al. [21]	Randomized, open-label phase 3	405	Nivolumab	3
Weber et al. [22]	Randomized, open label, phase 2	140	Nivolumab Ipilimumab	3; 3

### Chart audit

Among the 37 patients, 23 received Pembrolizumab, three Ipilimumab and 11 received both immunotherapies in sequence. All patients received at least one dose of immunotherapy. In total, the 37 patients received 48 treatments which were included in evaluation of AEs. None of the patients received combination immunotherapy.

### Product information

One additional AE, injection site reaction, was identified as being very common from the European Medicines Agency (EMA) product information on the three drugs. The AE had also occurred in one of the articles, however, since it was only identified in one data source and not being very common, it had not been initially included.

#### Adverse events to be included in the PRO-CTCA for melanoma patients

After thorough investigation of the literature, patients´ medical records and the product information 28 AEs were identified as relevant to include from the PRO-CTCAE-library (Table 2). Overall, there was great conformity between the three data sources.

**Table 2:** Adverse events included in the PRO-CTCAE for melanoma patients receiving immunotherapy based on findings in medical records, literature review and product information

Frequency of Adverse Events	CTCAE terms	Literature review	Medical records	Product information	PRO-CTCAE symptom terms
Very common (may affect more than 1 in 10 people)	*Vomiting*	*X*		*x*	*Vomiting*
	*Nausea*	*x*	*x*	*x*	*Nausea*
	*Anorexia*	*x*	*x*	*x*	*Decreased appetite*
	*Diarrhea*	*x*	*x*	*x*	*Diarrhea/Loose or watery stool*
	*Abdominal pain*	*x*	*x*	*x*	*Abdominal pain*
	*Constipation*	*x*		*x*	*Constipation*
	*Rash*	*x*	*x*	*x*	*Rash*
	*Pruritus*	*x*	*x*	*x*	*Itching*
	*Dyspnea*	*x*			*Shortness of breath*
	*Myalgia*	*x*	*x*		*Muscle pain*
	*Arthralgia*	*x*	*x*	*x*	*Joint pain*
	*Fatigue*	*x*	*x*	*x*	*Fatigue*
	*Injection site reaction*			*x*	*Pain and swelling at injection site*
	*Headache*	*x*	*x*		*Headache*
	*Chills*	*x*	*x*		*Chills*
	*Asthenia*	*x*		*x*	
Common (may effect up to 1 in 10 people	*Mucositis (oral)*	*x*	*x*	*x*	*Mouth/throat sores*
	*Dry skin*	*x*	*x*	*x*	*Skin dryness*
	*Alopecia*	*x*		*x*	*Hair loss*
	*Blurred vision*	*x*	*x*	*x*	*Blurred vision*
	*Cough*	*x*	*x*	*x*	*Cough*
	*Dysgeusia*	*x*	*x*	*x*	*Taste changes*
	*Dizziness*	*x*	*x*	*x*	*Dizziness*
	*Edema*	*x*		*x*	*Swelling*
	*Pain*	*x*		*x*	*General pain*
	*Peripheral sensory neuropathy*		*x*	*x*	*Numbness & tingling*
	*Hot Flashes*	*x*		*x*	*Hot flashes*
	*Flu –like symptoms*		*x*	*x*	
	*Pain in extremity*	*x*		*x*	
	*Back pain*	*x*		*x*	
Uncommon/not present	*Depression (2 items)*		*x*		*Discouraged Sad*
	*Blood in stool*				

Fifteen AEs were very common (may affect at least 10% of the patients). Eight of these *(nausea, anorexia, diarrhea, abdominal pain, rash, pruritus, arthralgia, fatigue)* were found common in all three data sources. The remaining seven (*vomiting, constipation, dyspnea, myalgia, injection site reaction, headache and chills*) were found very common in at least one or two of the data sources. The AE *asthenia* was also very common in both scientific papers and product information, however, since it was not found in the CTCAE or the PRO-CTCAE library, it was not included. This did not constitute a problem since the symptom was covered by the term fatigue which was found in the PRO-CTCAE-library. *Fatigue* is preferentially used in NCI’s toxicity grading scale that covers fatigue, asthenia, and malaise [26].

Eleven toxicities were found to be common (may affect up to 10% of patients) in two or three of the three data sources and were therefore included (Table 2). Other AEs which were also common such as *flu-like symptoms, pain in extremity,* and *back pain* were not included since they were not items in the PRO-CTCAE library. These terms seem adequately covered by muscle pain, joint pain, chills and the more general AE pain and thus, it was justifiable to exclude them.

Although the AE *depression* was uncommon [19] it was included as it was the only symptom dealing with mental health. Albeit rare, these symptoms can become very severe. Accordingly, two items from the PRO-CTCAE library concerning depression were selected. Despite the fact that the toxicity *blood in stool* was neither present in the PRO-CTCAE nor common, it was included as it may be a sign of colitis, a severe immune-related AE [27]. The question was placed at the end of the questionnaire as it was not a PRO-CTCAE item.

At face value, the questionnaire appeared to be a good instrument that adequately covered the relevant adverse events. Moreover, filling it out seemed to be uncomplicated and quick.

## Discussion

It was found that the AEs identified in the chart audit were consistent with the ones found in the literature search and the product information. This indicates that the information collected from these three sources was representative for melanoma patients receiving immunotherapy and usable when selecting relevant symptoms from the PRO-CTCAE library. The three information sources provided a clear picture of which symptoms to include in a questionnaire. Based on these findings, 28 PRO-CTCAE symptoms have been selected comprising of 56 PRO-questions plus an additional question on *blood in stool*.

It may be argued that having to leave a few symptoms out because they are not present in the PRO-CTCAE-library is a limitation. On the other hand, it is our belief that as long as these items are adequately covered by other items, the decision is justifiable. As the two PRO-CTCAE items dealing with depression can become very severe and may be hard to detect in a consultation at the outpatient clinic, it is vital that they are discovered as they arise. Increased attention, for example through frequent patient reporting, may be the way forward. When the study was designed, it was discussed whether or not to carry out focus group interviews with patients to further qualify the selection of items. We decided against it due to the fact it would not be possible to include all the experienced AEs anyway, however releveant to the individual patient. The same experience has been reported in other cancer poulations [28].

A special challenge in AE registration within immunotherapy may be that some symptoms occur rarely but can be life-threatening if detected too late. This may be an argument for including less frequent symptoms. On the other hand, a subtle balance exists between including the relevant symptoms while at the same time not exhausting the patients with too many questions [29]. In a previous study, it has been shown that a questionnaire containing a similar number of PRO-CTCAE questions (41 questions reflecting 22 symptomatic toxicities) has proven feasible in a Danish prostate cancer population receiving chemotherapy [15]. The patients found the questionnaire easy to fill out and not too time consuming (mean < 7 min.). In addition, 40% reported that it increased their focus on side effects. Others have demonstrated similar results [12] which supports the clinical feasibility of our suggested questionnaire for melanoma patients. Moreover, the fact that patients have the opportunity of adding other symptoms decreases the risk of infrequent AEs not being reported. When designing the study, it could have been considered to include a generic questionnaire that also deals with patients´ health related QoL such as the EORTC QLC-30 or the EQ-5D – used in most melanoma studies [30]. However, the present study focuses on detecting AEs early, and the PRO-CTCAE is specifically developed to enable patients to report on experienced AEs. Based on findings from Basch et al. [31] demonstrating improvement of QoL following PRO as intervention future studies should be designed with the inclusion of Qol measurement.

Furthermore, the material included in our analysis could be perceived as too comprehensive. Additional research has been warranted, however, to qualify the selection ofPRO-CTCAE items for given populations and contexts [32]. It was the purpose of this study to develop a PRO-CTCAE questionnaire for use in patients with metastatic melanoma who are treated with immunotherapy in the future. Consequently, three of the ten articles included described studies using the combination of two immunotherapies. This treatment was not standard treatment at the time of the review, however, it was introduced in Denmark in 2017, justifying the inclusion of studies testing the combination. Additionally, an article dealing with Ipilimumab as adjuvant treatment was included because the toxicity profile was evaluated as being identical to the profile seen in metastatic disease. Thus, the questionnaire can also be used for melanoma patients who receive adjuvant therapy.

## Conclusion

When melanoma patients receive immunotherapy, close monitoring of symptoms is crucial. One of the ways to detect AEs early may be to have the patients self-report the symptoms they experience, using PRO-questions. In this regard, it is important that the preparatory work to select questions is done properly. By going through the literature, examining the product information, and performing a chart audit, the most important and relevant symptoms have been uncovered, making it possible to design a PRO-questionnaire based on PRO-CTCAE that fits the patient population under investigation. This questionnaire is applied in an ongoing randomized clinical trial (ClinicalTrials.gov. NCT03073031) where melanoma patients treated with immunotherapy self-report the symptoms they experience.

## Abbreviations

AEs: Adverse events
CTCAE: Common Terminology Criteria for Adverse Events
EMA: European Medicines Agency
PROs: Patient-reported Outcomes
QoL: Quality of life
